# The T allele of TCF7L2 rs7903146 is associated with decreased glucose tolerance after bed rest in healthy older adults

**DOI:** 10.1038/s41598-022-10683-1

**Published:** 2022-04-27

**Authors:** Jean L. Fry, Brooke D. Munson, Katherine L. Thompson, Christopher S. Fry, Douglas Paddon-Jones, Emily J. Arentson-Lantz

**Affiliations:** 1 Department of Athletic Training and Clinical Nutrition, University of Kentucky, Lexington, KY 40536-0200 USA; 2 Dr. Bing Zhang Department of Statistics, University of Kentucky, Lexington, KY 40536-0082 USA; 3 Department of Nutrition & Metabolism, Center for Rehabilitation, Physical Activity and Nutrition, University of Texas Medical Branch, Galveston, TX 77555-1028 USA

**Keywords:** Genetics research, Translational research, Genetic association study, Predictive markers, Endocrinology, Metabolic syndrome, Pre-diabetes, Risk factors

## Abstract

Inpatient populations are at increased risk of hyperglycemia due to factors such as medications, physical inactivity and underlying illness, which increases morbidity and mortality. Unfortunately, clinicians have limited tools available to prospectively identify those at greatest risk. We evaluated the ability of 10 common genetic variants associated with development of type 2 diabetes to predict impaired glucose metabolism. Our research model was a simulated inpatient hospital stay (7 day bed rest protocol, standardized diet, and physical inactivity) in a cohort of healthy older adults (n = 31, 65 ± 8 years) with baseline fasting blood glucose < 100 mg/dL. Participants completed a standard 75 g oral glucose tolerance test (OGTT) at baseline and post-bed rest. Bed rest increased 2-h OGTT blood glucose and insulin independent of genetic variant. In multiple regression modeling, the transcription factor 7-like 2 (*TCF7L2) rs7903146* T allele predicted increases in 2-h OGTT blood glucose (p = 0.039). We showed that the *TCF7L2 rs7903146* T allele confers risk for loss of glucose tolerance in nondiabetic older adults following 7 days of bed rest.

## Introduction

Nondiabetic patients who develop hyperglycemia during hospitalization have increased lengths of stay and mortality risk^1–7^. In critically ill patients, mortality increases incrementally with rising blood glucose, and patients reaching values above 300 mg/dL have high mortality rates independent of diabetes diagnosis^3^. Likewise, postoperative hyperglycemia increases the risk of post-operative infection by 30% with every 40-point increase from normoglycemia (< 100 mg/dl)^7^. Currently, clinicians are unable to identify nondiabetic inpatients at greater risk for developing hyperglycemia, limiting their ability to initiate preventive therapy.

Dozens of common genetic variants are associated with increased risk for type 2 diabetes. For example, the odds of developing type 2 diabetes is 1.5 when having the transcription factor 7-like 2 (*TCF7L2*) *rs7903146* T allele (*rs7903146*^*T*^), which is established across many ethnic groups^8,9^. The *TCF7L2 rs7903146*^*T*^ variant is also associated with impaired pancreatic function and elevated glycated hemoglobin in nondiabetic individuals^10,11^. Though some genetic variants, including *TCF7L2 rs7903146*^*T*^*,* are associated with elevated glycemic indicators in nondiabetic individuals, more evidence is needed to determine how genetic testing of these risk variants may support clinical decision making in the inpatient setting.

Inpatient hyperglycemia is multifactorial and mediated by factors like physical inactivity, medications, medical nutrition therapies, and underlying acute illnesses/chronic disease^2,4,5,12^. Inpatient bed rest in healthy research subjects models the physical inactivity aspect of a hospital stay while avoiding the confounding influence of variable nutrition therapies and disease-related comorbidities. Therefore, inpatient bed rest is a powerful tool to understand how the interaction of physical inactivity and genetic variation may contribute to a decline in insulin sensitivity and subsequent impaired glucose tolerance independent of the catabolic burden of the clinical milieu.

We sought to determine if genetic testing for ten common type 2 diabetes risk variants could predict changes in fasting blood glucose or glucose intolerance during an oral glucose tolerance test (OGTT) challenge in healthy older adults completing a 7 day inpatient bed rest protocol. We selected genetic variants based on their previously reported effect size, effect on beta cell function, or location of the single nucleotide polymorphism (SNP), with preference for those located in exons^9,13,14^. Here we report on the ability *of MTNR1B (rs10830963), NOTCH2 (rs10923931), RASGRP1 (rs7403531), PROX1 (rs2075423), HHEX (rs1111875), IGF2BP2 (rs4402960), CDKAL1 (rs7754840), SLC30A8 (rs13266634), ZFAND6 (rs11634397), and TCF7L2 (rs7903146)* to predict changes in fasting blood glucose, 2-h OGTT blood glucose, and Matsuda Insulin Sensitivity Index (Matsuda-ISI)^15^ in 31 healthy older adults following a 7 day bed rest protocol.

## Results

### Participants

Thirty-one generally healthy older adults provided blood for genotyping and completed the inpatient bedrest protocol. Participants’ baseline characteristics are reported in Table 1.

**Table 1:** Participant demographics and baseline characteristics.

Characteristic	Value^a^
Sex (males/females; [%])	18/13 (58% / 42%)
Age (years)	65 ± 8
BMI^b^ (kg/m^2^)	26.9 ± 2.9
Systolic blood pressure (mmHg)	129 ± 15
Diastolic blood pressure (mmHg)	75 ± 8
Total cholesterol	187.3 ± 37.1
Triglycerides (mg/dL)	131.9 ± 45.6
HDL (mg/dL)	47.5 ± 10.8
HDL-C (mg/dL)	4.1 ± 1.0
LDL (mg/dL)	112.71 ± 35.0
VLDL	26.87 ± 7.7
Fasting insulin (μU/mL)	5.0 ± 3.6
Fasting glucose (mg/dL)	82.5 ± 6.4
Ethnicity (number: [%])	Caucasian: n = 21 (68%) Hispanic: n = 4 (13%) Black: n = 4 (13%) Asian: n = 2 (6%)

^1^Values are presented as mean ± standard deviation.

*BMI* body mass index.

### Overall effect of bed rest

**Figure 1:**
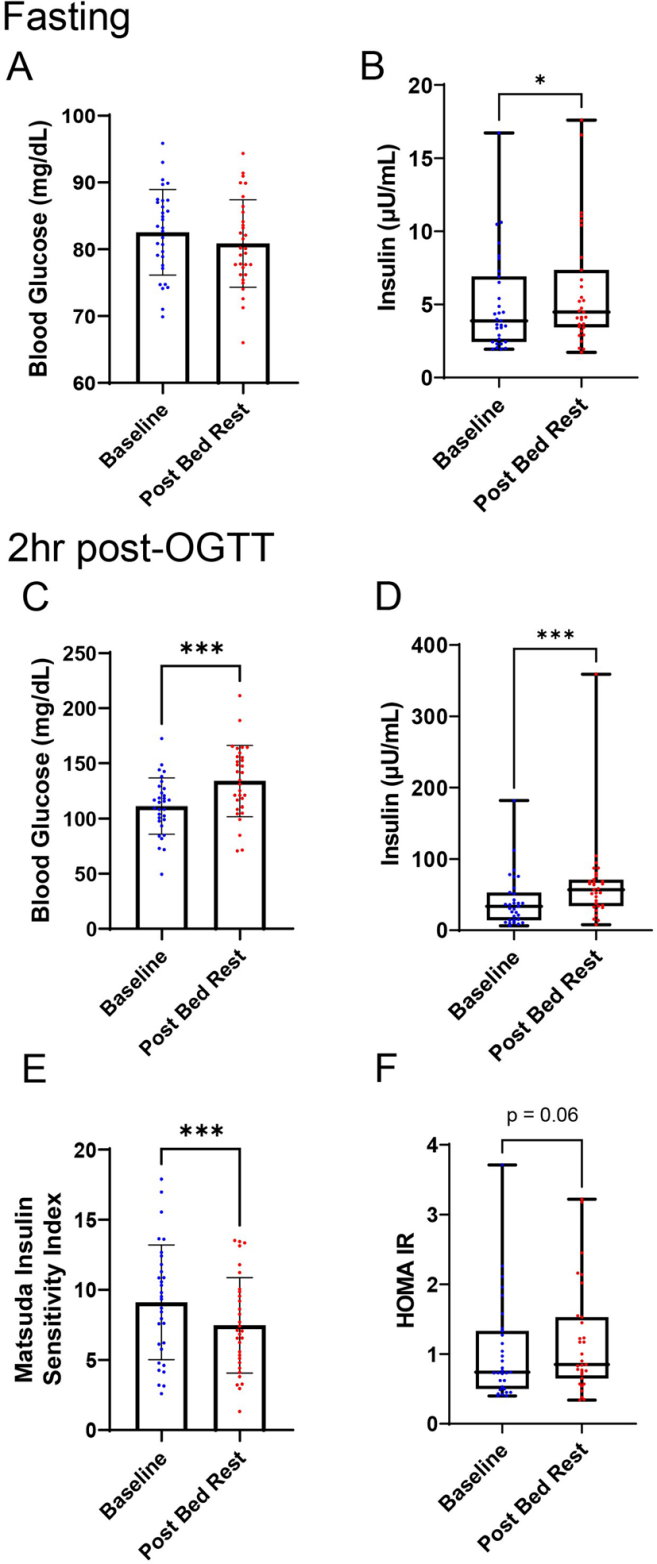
Overall glycemic responses to a 7-day inpatient bed rest protocol. (**a**) Fasting blood glucose before and after bed rest. (**b**) Fasting serum insulin before and after bed rest (**c**) 2 h post-OGTT blood glucose before and after bed rest (**d**) 2 h post-OGTT serum insulin before and after bed rest. (**e**) Matsuda Insulin Sensitivity Index (Mat-ISI) before and after bed rest. (**f**) Homeostatic Model Assessment for Insulin Resistance (HOMA-IR) before and after bed rest. *p-value < 0.05; **p-value < 0.01; ***p-value < 0.001. P-values are the result of paired t-test showing mean and standard deviation for variables meeting the assumptions (fasting blood glucose, 2 h post-OGTT blood glucose, and Matsuda Insulin Sensitivity Index); results of Wilcoxon Signed-Rank Tests showing median and interquartile range are shown for variables violating one or more assumptions (fasting insulin, 2 h post-OGTT insulin, and HOMA-IR).

Baseline and 2-h OGTT serum insulin and 2-h OGTT blood glucose were significantly increased in healthy older adults following seven days of bed rest contributing to significantly decreased insulin sensitivity and increased insulin resistance as indicated by Matsuda-ISI and Homeostatic Model for Insulin Resistance (HOMA-IR^16^), respectively (Fig. 1). Complete overall glucose and insulin responses to OGTT before and after bed rest are reported in Supplementary Table S1.

### Models predicting increases in blood glucose

**Figure 2:**
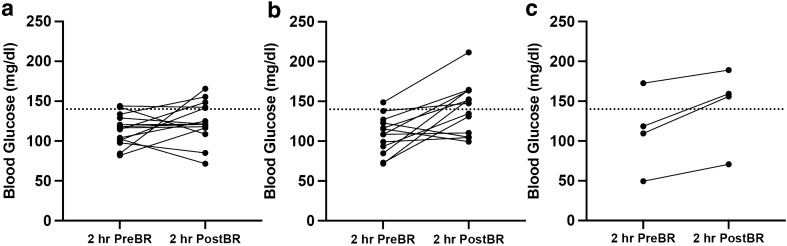
Spaghetti plot of blood glucose 2 h before (PreBR) and after (PostBR) a 7-day inpatient bed rest protocol by *TCF7L2 rs7903146*^*T*^ genotype (**a**) Individual 2-h OGTT blood glucose changes in C/C genotype group; (**b**) Individual 2-h OGTT blood glucose changes in C/T genotype group (**c**) Individual 2-h OGTT blood glucose changes in T/T genotype group; the dotted line on (**a**–**c**) is 140 mg/dl, which is the cut point for a normal 2-h blood glucose value of an OGTT. (p = 0.039 for partial F-test of overall model *TCF7L2 rs7903146*^*T*^ allele as a significant predictor of increased 2-h OGTT blood glucose following bed rest when including baseline 2-h OGTT blood glucose, age, and BMI as main effects in the model).

**Figure 3:**
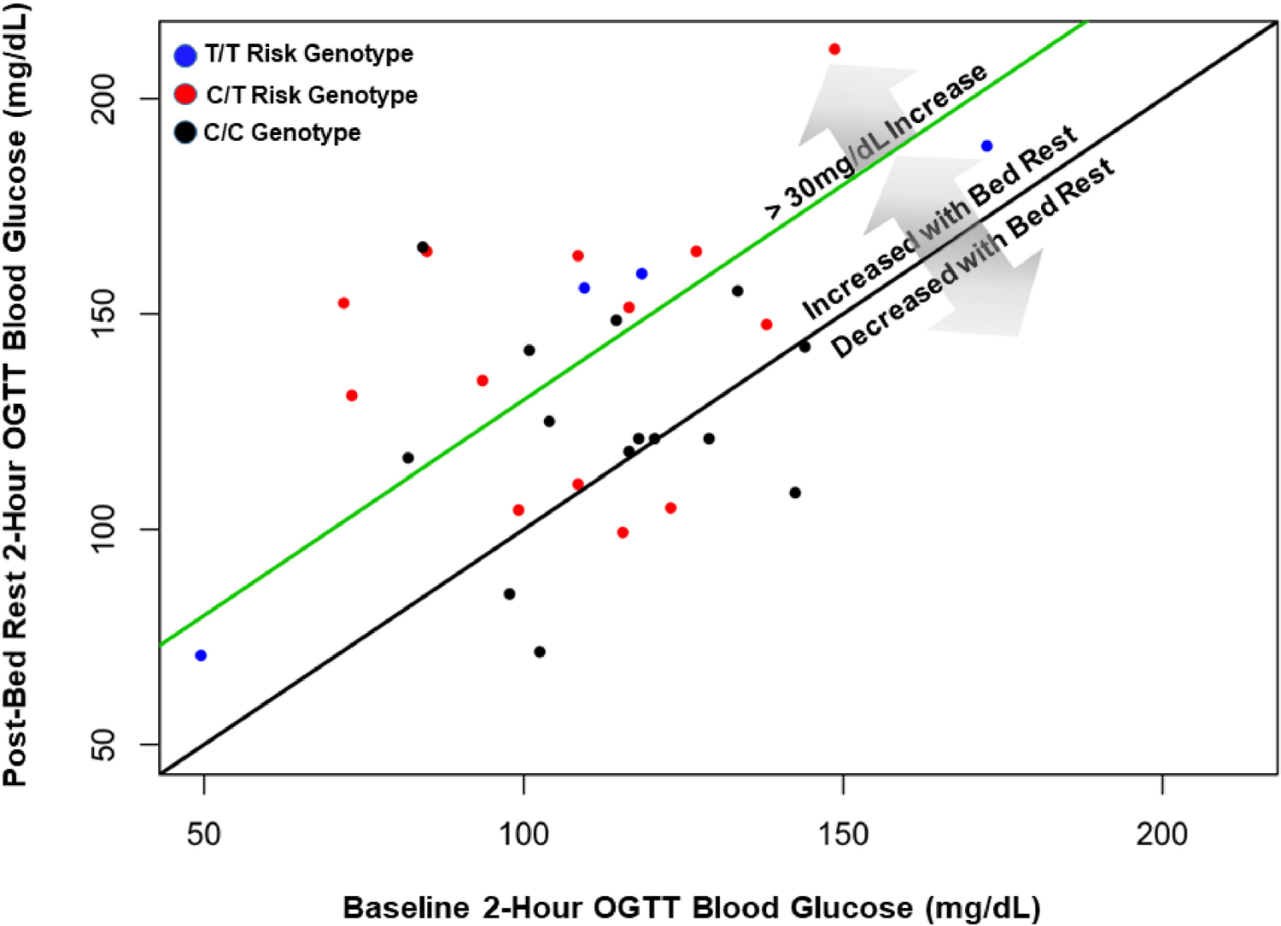
Association of PreBR 2-h OGTT blood glucose with PostBR 2-h OGTT blood glucose. The black line represents no change in 2-h OGTT glucose following 7 days of bed rest. The green line represents a 30 mg/dL increase in blood glucose after the 7 day bed rest protocol. Individuals with the C/C risk genotype are shown as black circles, C/T risk variants are red circles and T/T risk variants are blue circles. (p = 0.039 for partial F-test of overall model *TCF7L2 rs7903146*^*T*^ allele as a significant predictor of increased 2-h OGTT blood glucose following bed rest when including baseline 2-h OGTT blood glucose, age, and BMI as main effects in the model).

During model selection, the Feasible Solutions Algorithm^17–19^ identified the *TCF7L2 rs7903146*^*T*^ allele and its statistical interaction effect with baseline 2-h OGTT blood glucose as a significant predictor of increased 2-h OGTT blood glucose following bed rest when including baseline 2-h OGTT blood glucose, age, and BMI as main effects in the model (p = 0.039). The baseline 2-h OGTT blood glucose and the *TCF7L2 rs7903146*^*T*^ allele together explained 23.65% of the variability in post-bed rest 2-h OGTT blood glucose values, after controlling for age and BMI. (Figs. 2 and 3). There were no significant baseline differences in age, BMI, systolic blood pressure, diastolic blood pressure, fasting blood glucose or baseline 2-h OGTT blood glucose between *TCF7L2 rs7903146* genotype groups at study baseline (Supplementary Table S2). No other genotype predicted glycemic outcomes or Matsuda-ISI following seven days of bed rest.

## Discussion

Older adults with *TCF7L2 rs7903146*^*T*^ risk variants are more likely to have increased 2-h OGTT blood glucose following seven days of bed rest. No significant relationships where found for the 9 other variants, but OGTT data for all SNPs assessed is presented in Supplementary Tables S3 and S4.

TCF7L2 is a transcription factor belonging to the Wnt signaling pathway present in pancreas, liver, and other tissues^20,21^. Whole-genome chromatinimmunoprecipitation (ChIP) combined with massively parallel DNA sequencing (ChIP-Seq) analyses show that TCF7L2 binds directly to several genes involved in glucose metabolism, including *PCK1, FBP1, IRS1, IRS2, AKT2, ADIPOR1, PDK4 AND CPT1A*^22^. Carriers of the *rs7903146*^*T*^ allele exhibit impaired proinsulin conversion, higher proinsulin:insulin ratios, and greater likelihood of developing insulin-dependent type 2 diabetes^23–26^, but not hepatic or extrahepatic insulin resistance^27,28^. Paradoxically, some evidence indicates that liver and other tissues appear to be involved in *TCF7L2 rs7903146*^*T*^-associated glucose intolerance and insulin secretion^29^.

*TCF7L2* risk alleles are associated with elevated post-OGTT and nocturnal blood glucose in nondiabetic adults^25,26,30,31^. The *rs7903146*^*T*^ allele also associates with impaired glucose tolerance in adults with metabolic syndrome^30^ and obese adolescents^25^. Healthy, middle-aged and older nondiabetic participants with *rs7903146*^*T*^ also exhibit higher nocturnal glucose^31^. However, similar to our findings, prior studies indicate that *rs7903146*^*T*^ does not affect *fasting* blood glucose in healthy middle-aged adults^32^. This suggests that fasting blood glucose may not be an optimal biomarker to screen individuals with *rs7903146*^*T*^ for risk of developing prediabetes and type 2 diabetes.

Thirty-eight young, healthy Caucasian men with *TCF7L2 rs7903146*^*T*^ risk alleles exhibit a lower first-phase insulin response (FPIR) to an intravenous glucose tolerance test (IGTT) compared with those with the homozygous C genotype both before and after 9 days of bed rest (p = 0.01 and p = 0.0001, respectively)^33^. Following bed rest, the participants with the *TCF7L2 rs7903146* risk variants also fail to show an incremental rise of FPIR in response to insulin resistance. Though FPIR is not a concept that is directly translatable outside of the context of an IGTT, the ability to rapidly secrete insulin in response to an OGTT in the early phase (up to 30 min after consumption of glucose) is a similar concept^34^. The liver responds to a robust early phase insulin response by reducing release of glucose, thereby limiting the overall blood glucose response to an OGTT or a meal, and this physiological trait that is lost in the development of type 2 diabetes^35^. Here we did not observe any relationship between *rs7903146*^*T*^ risk alleles and insulin measures or calculated insulin sensitivity at any point after a 75 g glucose load, but we completed 2-h OGTT, which are not directly comparable to the IGTT or the FPIR.

Periods of physical inactivity promote insulin resistance in healthy adults^36,37^. If the reduced glucose tolerance we observed following a 7 day period of inactivity in healthy, nondiabetic adults persists, patients having *rs7903146*^*T*^ variants could be especially susceptible to long-term impairments in glucose metabolism following inpatient stays. Future research should evaluate how *rs7903146*^*T*^ affects blood glucose throughout hospital stay in both critically ill and non-critically ill hospital patients. Moreover, follow up studies should evaluate if *rs7903146*^*T*^ predicts long-term glucose intolerance following an extended period of disuse in clinical populations in patients after discharge. Finally, utilizing OGTT, rather than fasting blood glucose, may be more appropriate for patients carrying the *rs7903146*^*T*^* allele.*

Our analysis showed an intriguing association between the *TCF7L2 rs7903146*^*T*^ allele and loss of glucose tolerance after physical disuse; however, there were limitations. This study was not initially designed to test genotype–phenotype relationships. We recruited volunteers to test the effect of nutrition and physical activity on a broad range of outcomes following bed rest. Since parent study intervention groups did not associates with any glycemic outcome, and multivariate modeling did not identify parent study group assignment as a significant factor in the model, we feel confident that the genotype–phenotype relationship between *TCF7L2 rs7903146*^*T*^ and 2-h OGTT blood glucose described here was not affected by parent study group assignment. Since we only genotyped 10 variants, we could not consider any possible contribution of genetic background to the outcomes. Finally, this small study was conducted in generally healthy older adults and would not be generalizable to other aged groups or those with acute or chronic illness.

The *TCF7L2 rs7903146*^*T*^ genotype did not predict development of clinically diagnosable hyperglycemia (fasting blood glucose ≥ 126 mg/dl) or impaired glucose tolerance (2-h OGTT blood glucose ≥ 140 mg/dl) following bed rest. However, the findings from this study indicate that patients with the *TCF7L2 rs7903146*^*T*^ allele may be at risk for accelerated decline in glucose tolerance with bed rest and may benefit from closer glucose monitoring during hospitalization.

## Conclusion

We show for the first time that the TCF7L2 rs7903146 T allele associates with increased 2-h OGTT blood glucose in nondiabetic, older adults following seven days of bed rest and physical disuse. If these findings can be replicated in a clinical setting, the *TCF7L2 rs7903146*^*T*^ allele may help clinicians identify nondiabetic inpatients at greater risk for accelerated glucose intolerance and hyperglycemia in an inpatient setting.

## Methods

### Participants

Thirty-one healthy older adults were recruited (65 ± 8 years), provided written informed consent, medically screened, and compensated for their time as part of a larger randomized-controlled trial. A fasting glucose ≥ 100 mg/dl, recent corticosteroid use, or evidence of chronic disease (vascular disease, unmanaged elevated blood pressure, and kidney disease) were considered exclusionary criteria for the study. All enrolled participants were community-dwelling, able to complete activities of daily living, and considered to be generally healthy. The study protocol was conducted within the inpatient unit of the Clinical and Translational Research Center at the University of Texas Medical Branch (UTMB) and in accordance with the Declaration of Helsinki and approved by the UTMB Institutional Review Board. All participants provided informed consent including consent for genetic analyses. Sample size was determined by subject enrollment in the parent study and available blood samples for genetic analyses. Recruitment and collection of blood for this secondary analysis began after the initial enrollment period for the parent clinical trial. Participants included in the present analysis were enrolled between 03/26/2014 and 10/10/2017, the latter of which was the last enrollment for the parent grant. This study was registered though clinicaltrials.gov on 03/05/2013 (NCT01846130).

Participants were assigned to one of five experimental conditions (protein consumption patterns, small bout of walking, or amino acid supplementation)^38–40^ that were outside of the scope of this study. Collection and storage of blood for genotyping was initiated in the middle of the parent grant, so not all study participants could be included in the analysis. The study statistician confirmed that there were no significant relationships between the study interventions and primary study outcomes (2-h OGTT glucose and Matsuda-ISI) presented here (p > 0.05). A brief description of each study intervention is available in Supplementary Table S5.

### Bed rest

**Figure 4:**
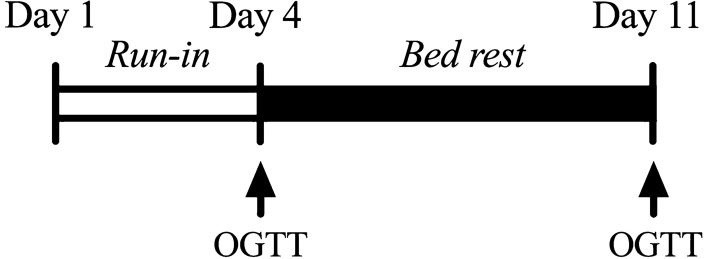
Overall study design: Nondiabetic participants spent 2 inpatient days completing a period of diet stabilization before undergoing a baseline oral glucose tolerance test (OGTT), followed by seven days of bed rest, and a post-bed rest OGTT.

As previously reported^38–40^ participants completed 3 days of diet-stabilization/testing followed by seven days of horizontal bed rest in the UTMB Institute for Translational Sciences–Clinical Research Center (ITS-CRC). Consistent with our previous horizontal bed rest studies, subjects were continuously monitored for safety^41^. All bathing and toiletry activities were performed without bearing weight. The general experimental design is depicted in Fig. 4.

### Diet

Participants were provided isoenergetic diets (55% carbohydrate, 29% fat, and 16% protein). Individualized daily energy requirements were estimated using the Harris–Benedict equation with activity factors of 1.6 and 1.3 used for the ambulatory and bed rest period, respectively^38,39,41^. Water was provided ad libitum. The breakfast meal presented after each OGTT was adjusted to compensate for the 75 g glucose load. Energy and macronutrient intake, taking plate waste into account, were analyzed by using Nutrition Data System for Research software (version 2011, Nutrition Coordinating Center, Minneapolis, MN).

### Oral glucose tolerance test and serum insulin

Standard 75 g glucose load (Glucola, Azer Scientific, Morgantown, PA) oral glucose tolerance tests were administered before and after the 7-day bed rest protocol. Whole-blood samples (0, 30, 60, 90, and 120 min) were analyzed on an YSI Bioanalyzer (YSI, Yellow Springs, OH). Serum insulin was measured using a commercially available enzyme-linked immunosorbent assay (MilliporeSigma, Burlington, MA). Matsuda Insulin Sensitivity Index (Matsuda-ISI) was determined using the Matsuda formulas^15^.

### SNP selection and genotyping

Though dozens of genetic variants have been reported to associate with diabetes and other metabolic conditions, we chose an initial subset of SNPs showing a relatively large effect size, a demonstrated impact on beta cell function, and/or evidence of translated protein products in older adults^9,13,14^.

Genomic DNA was extracted from whole blood samples using the DNeasy Blood Kit (QIAGEN, Germantown, MD) according to the manufacturer's instructions. TaqMan® Genotyping Assays (Thermo Fisher/Applied Biosystems, Foster City, CA) were used to genotype for *MTNR1B (rs10830963), NOTCH2 (rs10923931), RASGRP1 (rs7403531), PROX1 (rs2075423), HHEX (rs1111875), IGF2BP2 (rs4402960), CDKAL1 (rs7754840), SLC30A8 (rs13266634), ZFAND6 (rs11634397), and TCF7L2 (rs7903146)* (Supplementary Tables S6 and S7). The work was performed by the Genomics Core at the University of Texas Medical Branch according to the manufacturer’s instructions. Briefly, 5 ng of purified DNA was added to each well and dried down. Primers and all reagents were combined, and PCR was completed using an Applied Biosystems 7500 Fast Real-Time PCR System (Thermo Fisher/Applied Biosystems, Foster City, CA). Thereafter, Sequence Detection System (SDS) Software was used to perform a post-PCR plate read and analysis to call genotypes. Samples were run in triplicate and the core was able to make genotype calls for all participants and single nucleotide polymorphisms (SNP). Genotyping assays were completed at the same time using reagents from the same lot and were carried out by the same technician. To confirm accurate genotype calls, the genomics core demonstrated concordance with the known genotypes for Hela, HEK, HEMS and two other known reference samples. After statistical analyses revealed significant associations between the associations between the TCF7L2 rs7903146 genotype and change in glucose tolerance with best rest, we confirmed the genotyping results by genotyping for TCF7L2 rs4506565 which is in linkage disequilibrium with rs7903146^42,43^.A TaqMan® Genotyping Assay was completed using the methods described above on a QuantStudio™ Fast Real-Time PCR System (Thermo Fisher/Applied Biosystems, Foster City, CA) in the Center for Muscle Biology at the University of Kentucky. Allele frequencies for *TCF7L2 (rs7903146) and* TCF7L2 rs4506565 from this study and other US groups are included in Supplementary Table S8^44^. All genotyping results can be viewed in Supplementary Data File 1. The allelic discrimination plot for TCF7L2 rs7903146 provided by the UTMB Genomics Core is included in Supplementary Data File 2.

### Statistical analyses

First, summary statistics were calculated for demographic and baseline characteristics. In particular, means and standard deviations were calculated for quantitative variables, while percentages were calculated for categorical variables. Genotype frequencies were calculated for each SNP, and Hardy Weinberg Equilibrium exact tests were also performed using the R package, HardyWeinberg^45^.

Prior to analysis of the primary outcome (fasting glucose, 2-h OGTT glucose) and the secondary outcome (Matsuda-ISI), a one-way ANOVA was run to screen for any significant baseline differences with each parent study intervention and genotype groups. Overall pre-post bed rest comparisons of quantitative variables were made using paired t-tests or Wilcoxon Signed-Rank tests depending on the result of a Shapiro-Wilks test on the pre-post differences in quantitative variables.

Statistical model selection^46^ was performed to identify multiple linear regression models that are able to predcit blood glucose and Matsuda score^32^. In particular, we were interested in identifying explanatory variables that improved predictions of each outcome after accounting for age, BMI, and baseline value of the outcome (if the baseline value was collected). Since adjustments were required, we used the Feasible Solutions Algorithm^17,19^ to generate candidate multivariate regression models for each outcome, using R^2^ as the statistical criterion for selection. Potential explanatory variables included all combinations of two of the 10 candidate SNPs, age, BMI, and/or parent study intervention.

The Feasible Solutions Algorithm identified candidate multiple linear regression models, from which a model was selected for each outcome variable. For the final multiple linear regression model selected, a partial F-test was performed to look for evidence that the identified SNP was helpful in predicting the post-time point primary outcome, after accounting for age, BMI, and baseline outcome value (as appropriate). For OGTT, a scatterplot was created to show the relationship between pre-BR OGTT and post-OGTT values observed in the data. Color of observed data point is used to distinguish TCF7L2 rs7903146 variants (Fig. 3). All analyses were performed in R version 3.6.1.

### Data availability

The datasets generated during and/or analyzed during the current study are available from the corresponding author on reasonable request.

## Supplementary Information

Supplementary Information 1.

Supplementary Information 2.

Supplementary Information 3.

Supplementary Information 4.
