# Supplementary figures and images for: The T allele of TCF7L2 rs7903146 is associated with decreased glucose tolerance after bed rest in healthy older adults
Source: Sci Rep. 2022 Apr 27;12:6897. doi: 10.1038/s41598-022-10683-1 (PMC9046412; doi:10.1038/s41598-022-10683-1)

**Allelic Discrimination GCM1611-1**


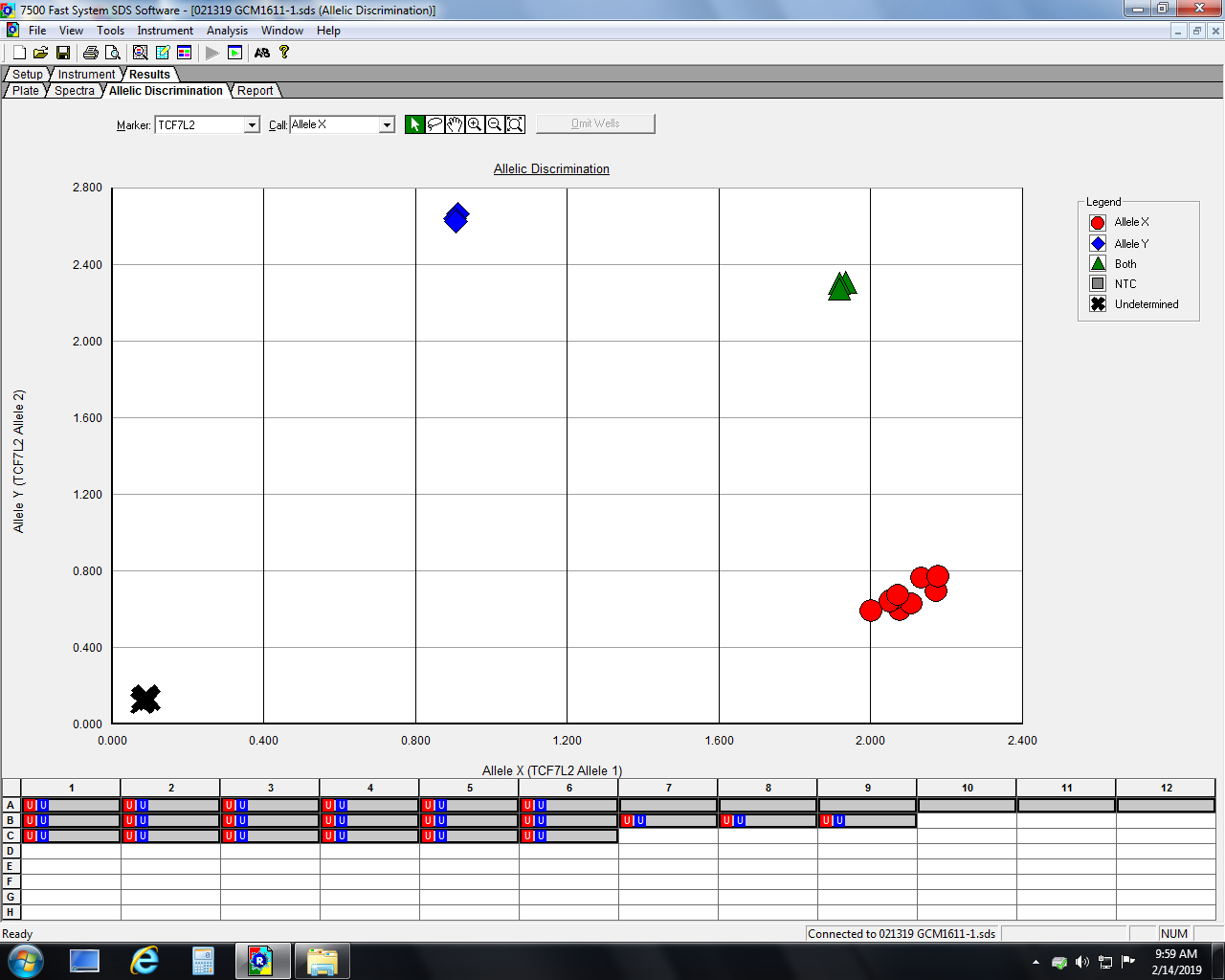


**2/13/19**

Supplement: Supplementary file 2 — Supplementary Information 2. [file 41598_2022_10683_MOESM2_ESM.docx]
