# Supplementary material for: The T allele of TCF7L2 rs7903146 is associated with decreased glucose tolerance after bed rest in healthy older adults
Source: Sci Rep. 2022 Apr 27;12:6897. doi: 10.1038/s41598-022-10683-1 (PMC9046412; doi:10.1038/s41598-022-10683-1)
Supplement: Supplementary file 3 — Supplementary Information 3. [file 41598_2022_10683_MOESM3_ESM.doc]

**
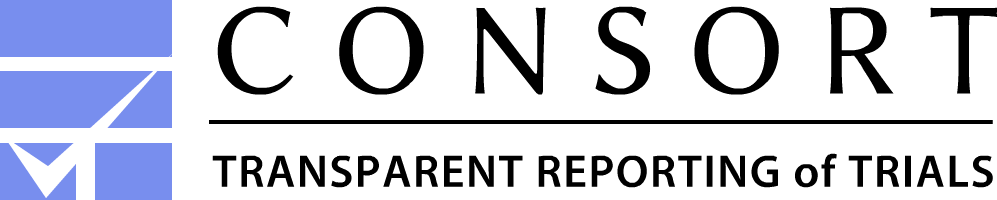
**

**CONSORT 2010 Flow Diagram**

**Allocation**

**Analysis**

**Enrollment**

Telephone Screened (n=374)

Medical Screening 1 (n=123)

Medical Screening 2 (n=64)

Excluded (n= 70)

♦  First Screening (n=54)

♦  Second Screening (n=8)

♦  Declined to participate (n=8)

Allocated to CON (n=11 )

♦ Received allocated intervention (n=10)

♦ Did not receive allocated intervention (dropped out prior to beginning bed rest portion of the study due to unrelated illness) (n=1 )

Randomized (n=53)

Allocated to LEU (n=10)

♦ Received allocated intervention (n=10)

Allocated to WHEY (n=10)

♦ Received allocated intervention (n=10)

Allocated to STEP (n=10)

♦ Received allocated intervention (n=10)

Allocated to SKEW (n=12)

♦ Received allocated intervention (n=10)

♦Did not receive allocated intervention (dropped out prior to beginning bed rest portion of the study due to unrelated personal issues (n=2 )

CON Analyzed (n=8)

♦ Excluded from analysis due to lack of sufficient blood sample (n=2)

LEU Analyzed (n=8)

♦ Excluded from analysis due to lack of sufficient blood sample (n=2)

WHEY Analyzed (n=8)

♦ Excluded from analysis due to lack of sufficient blood sample (n=2)

STEP Analyzed (n=1)

♦ Excluded from analysis due to lack of sufficient blood sample (n=9)

SKEW Analyzed (n=6)

♦ Excluded from analysis due to lack of sufficient blood sample (n=4)

Analyzed (n=31)
