# Supplementary material for: The T allele of TCF7L2 rs7903146 is associated with decreased glucose tolerance after bed rest in healthy older adults
Source: Sci Rep. 2022 Apr 27;12:6897. doi: 10.1038/s41598-022-10683-1 (PMC9046412; doi:10.1038/s41598-022-10683-1)
Supplement: Supplementary file 4 — Supplementary Information 4. [file 41598_2022_10683_MOESM4_ESM.pdf]

Supplementary Table S1. Overall effect of seven days of bed rest on participant glycemic indicators (n=31)

	Before Bed Rest		After Bed Rest	
OGTT[1] Time Point	Blood Glucose (mg/dL, Mean ± SD)	Serum Insulin (μU/mL, Mean ± SD)	Blood Glucose (mg/dL, Mean ± SD)	Serum Insulin (μU/mL, Mean ± SD)
0 min	82.5 ± 6.4	5.0 ± 3.4	80.8 ± 6.5	5.9 ± 4.0
30 min	135.8 ± 24.4	43.5 ± 29.5	133.2 ± 18.4	56.0 ± 69.1
60 min	142.2 ± 30.7	45.4 ± 32.3	149.5 ± 26.4	62.4 ± 72.7
90 min	130.6 ± 27.1	54.6 ± 55.1	145.8 ± 28.3	63.1 ± 65.5
120 min	111.2 ± 25.4	41.6 ± 36.9	134.1 ± 32.2	63.8 ± 60.0

[1] OGTT: Oral Glucose tolerance test; blood glucose and insulin values were assessed in 30 min intervals following a 75 g glucose challenge

Supplementary Table S2. Baseline characteristics by TCF7L2 rs7903146 genotype.

	TCF7L2 rs7903146 C/C (n=14)	TCF7L2 rs7903146 C/T (n=13)	TCF7L2 rs7903146 T/T (n=4)
Age (years)	66.4 ± 8.7	64.6 ± 7.6	64 ± 9.8
BMI[1]	26.9 ± 2.7	26.7 ± 3.2	27.8 ± 3
SBP[2]	1278 ± 12	130 ± 17	131 ± 22
DBP[3]	74 ± 5	74 ± 10	81 ± 4
FBG[4]	81.9 ± 4.8	83 ± 6.7	83.5 ± 11.4
2-hour OGTT[5]	113.6 ± 19.5	108.3 ± 23.5	112.5 ± 50.4

[1] BMI: Body mass index

[2] SBP: Systolic blood pressure in mmHg

[3] DBP: Diastolic blood pressure in mmHg

[4] FBG: fasting blood glucose in mg/dL

[5] 2-hour OGTT: Blood glucose in mg/dL 2 hours after 75 g oral glucose tolerance test at study baseline

Supplementary Table S3. Glucose responses to 75 g oral glucose tolerance test (OGTT) before and after 7 days of bed rest by genotype.

	OGTT Glucose PreBR[1] (mg/dL; mean ± SD)			OGTT Glucose PostBR (mg/dL; mean ± SD)		
	TCF7L2 rs7903146 C/C (n=14)	TCF7L2 rs7903146 C/T (n=13)	TCF7L2 rs7903146 T/T (n=4)	TCF7L2 rs7903146 C/C (n=14)	TCF7L2 rs7903146 C/T (n=13)	TCF7L2 rs7903146 T/T (n=4)
0 min	81.9 ± 4.8	83 ± 6.7	83.5 ± 11.4	79.8 ± 5.3	82 ± 6.3	80.7 ± 11.7
30 min	135.2 ± 28.8	137.1 ± 21.7	133.7 ± 20.8	139.1 ± 16.2	134.1 ± 20.9	141.2 ± 18.6
60 min	144.4 ± 32.9	142.1 ± 21.8	135.3 ± 52.2	140.4 ± 20.9	154.5 ± 29.7	165.6 ± 26.4
90 min	129.3 ± 26.1	133.3 ± 20.75	130.6 ± 27.1	139.6 + 19.6	149 ± 35.5	157.4 ± 30.3
120 min	113.6 ± 19.5	108.3 ± 23.5	112.5 ± 50.4	124.3 ± 25.7	141.6 ± 32	143.8 ± 50.9
	MTNR1B rs10830963 C/C (n=15)	MTNR1B rs10830963 C/G (n=15)	MTNR1B rs10830963 G/G (n=1)	MTNR1B rs10830963 C/C (n=15)	MTNR1B rs10830963 C/G (n=15)	MTNR1B rs10830963 G/G (n=1)
0 min	81.9 ± 6.5	82.3 ± 5.7	95.9 ± 0	80.3 ± 6.6	80.6 ± 5.9	94.4 ± 0
30 min	130.5 ± 18.4	141.5 ± 29.4	129.0 ± 0	133.7 ± 14.3	131.1 ± 21.6	158.0 ± 0
60 min	138.1 ± 31.9	146.3 ± 31.0	143.0 ± 0	148.7 ± 17.9	149.0 ± 33.7	170.3 ± 0
90 min	129.8 ± 31.9	131.1 ± 23.4	134.5 ± 0	145.9 ± 27.8	143.4 ± 29.1	181.5 ± 0
120 min	116.8 ± 29.8	105.2 ± 20.5	118.5 ± 0	137.4 ± 36.1	129.0 ± 28.8	159.3 ± 0

	NOTCH2 rs1092393 G/G (n=26)	NOTCH2 rs1092393 G/T (n=4)	NOTCH2 rs1092393 T/T (n=1)	NOTCH2 rs1092393 G/G (n=26)	NOTCH2 rs1092393 G/T (n=4)	NOTCH2 rs1092393 T/T (n=1)
0 min	82.6 ± 6.7	80.9 ± 5.4	87.5 ± 0	80.9 ± 6.6	81.6 ± 7.7	77.7 ± 0
30 min	136.7 ± 25.4	136.9 ± 17.3	108.0 ± 0	132.9 ± 19.5	137.9 ± 10.4	121.5 ± 0
60 min	139.3 ± 32.5	158.0 ± 11.1	156.0 ± 0	148.1 ± 25.9	152.7 ± 33.4	175.0 ± 0
90 min	126.1 ± 27.2	153.1 ± 8.3	156.0 ± 0	141.8 ± 26.2	158.1 ± 30.0	202.0 ± 0
120 min	108.6 ± 26.4	118.6 ± 8.5	148.7 ± 0	131.6 ± 30.6	130.9 ± 21.8	211.5 ± 0
	RASGRP1 rs74035 C/C (n=21)	RASGRP1 rs74035 C/T (n=7)	RASGRP1 rs74035 T/T (n=3)	RASGRP1 rs74035 C/C (n=21)	RASGRP1 rs74035 C/T (n=7)	RASGRP1 rs74035 T/T (n=3)
0 min	82.0 ± 6.7	85.4 ± 5.1	79.6 ± 6.9	80.4 ± 6.9	82.8 ± 6.8	79.6 ± 2.6
30 min	138.4 ± 23.4	135.9 ± 30.6	117.7 ± 6.3	133.7 ± 19.3	128.7 ± 19.8	140.2 ± 2.0
60 min	146.8 ± 30.3	139.8 ± 26.0	115.7 ± 40.3	155.7 ± 28.2	135.5 ± 17.8	139.1 ± 16.3
90 min	132.6 ± 25.3	134.8 ± 24.4	106.5 ± 42.7	148.8 ± 31.2	140.0 ± 21.7	138.5 ± 23.6
120 min	113.5 ± 23.8	113.9 ± 25.5	89.0 ± 35.1	135.5 ± 34.3	140.0 ± 22.4	110.1 ± 36.1
	PROX1 rs2075423 T/T (n=6)	PROX1 rs2075423 G/T (n=11)	PROX1 rs2075423 G/G (n=14)	PROX1 rs2075423 T/T (n=6)	PROX1 rs2075423 G/T (n=11)	PROX1 rs2075423 G/G (n=14)
0 min	86.3 ± 5.4	83.6 ± 6.3	80.1 ± 6.3	83.8 ± 8.5	80.6 ± 5.4	79.9 ± 6.6
30 min	149.9 ± 20.7	138.0 ± 27.1	128.0 ± 21.9	139.7 ± 20.3	128.6 ± 18.9	134.0 ± 17.5
60 min	146.5 ± 23.8	138.1 ± 24.5	143.6 ± 38.3	170.6 ± 27.6	140.0 ± 24.0	148.0 ± 24.1
90 min	121.8 ± 20.5	135.4 ± 23.6	130.5 ± 32.3	158.4 ± 29.5	135.8 ± 18.4	148.4 ± 33.0

120 min	107.4 ± 9.1	113.6 ± 23.2	111.0 ± 32.2	143.0 ± 19.2	130.0 ± 18.6	133.5 ± 43.9
	HHEX rs111187 T/T (n=4)	HHEX rs111187 C/T (n=13)	HHEX rs111187 C/C (n=14)	HHEX rs111187 T/T (n=4)	HHEX rs111187 C/T (n=13)	HHEX rs111187 C/C (n=14)
0 min	75.8 ± 5.2	83.4 ± 5.3	83.7 ± 6.8	76.2 ± 7.0	82.1 ± 4.6	81.1 ± 7.7
30 min	131.1 ± 22.0	135.8 ± 26.4	137.1 ± 24.6	128.3 ± 17.7	137.6 ± 12.1	130.5 ± 23.1
60 min	150.5 ± 39.3	139.8 ± 28.0	142.1 ± 32.6	145.8 ± 12.4	151.1 ± 25.6	149.2 ± 30.9
90 min	144.0 ± 26.9	129.2 ± 29.4	128.1 ± 25.7	151.8 ± 27.9	139.8 ± 26.1	149.7 ± 31.2
120 min	111.0 ± 44.9	115.3 ± 27.3	107.5 ± 17.4	151.6 ± 38.0	130.8 ± 35.1	132.1 ± 28.5
	IGF2BP2 rs440296 G/G (n=11)	IGF2BP2 rs440296 G/T (n=17)	IGF2BP2 rs440296 T/T (n=3)	IGF2BP2 rs440296 G/G (n=11)	IGF2BP2 rs440296 G/T (n=17)	IGF2BP2 rs440296 T/T (n=3)
0 min	83.9 ± 6.6	82.0 ± 5.0	80.5 ± 13.4	84.8 ± 5.6	78.6 ± 4.3	79.4 ± 14.2
30 min	144.3 ± 20.3	132.6 ± 27.1	122.8 ± 14.8	145.6 ± 11.0	123.5 ± 16.3	142.3 ± 23.3
60 min	143.0 ± 23.9	139.5 ± 34.2	154.8 ± 39.6	162.6 ± 31.0	139.1 ± 20.8	160.4 ± 10.7
90 min	133.9 ± 22.6	126.9 ± 28.3	139.1 ± 41.8	153.3 ± 29.8	139.7 ± 26.7	153.2 ± 34.0
120 min	102.5 ± 20.4	114.6 ± 24.4	124.3 ± 45.6	140.3 ± 18.2	126.3 ± 37.4	154.9 ± 36.4
	CDKAL1 rs775484 G/G (n=10)	CDKAL1 rs775484 C/G (n=19)	CDKAL1 rs775484 C/C (n=2)	CDKAL1 rs775484 G/G (n=10)	CDKAL1 rs775484 C/G (n=19)	CDKAL1 rs775484 C/C (n=2)
0 min	83.0 ± 6.9	82.2 ± 5.9	83.9 ± 12.9	79.9 ± 7.3	81.0 ± 6.1	84.3 ± 9.4
30 min	137.1 ± 26.5	137.9 ± 23.5	109.2 ± 4.5	128.9 ± 24.8	134.5 ± 14.8	142.3 ± 15.9

60 min	146.7 ± 33.1	141.7 ± 31.2	125.0 ± 3.5	145.0 ± 32.0	154.2 ± 22.5	127.8 ± 30.1
90 min	124.4 ± 19.6	134.4 ± 30.2	125.4 ± 36.2	152.4 ± 31.9	145.9 ±26.0	112.3 ± 4.6
120 min	105.4 ± 16.5	117.8 ± 27.3	77.5 ± 6.2	132.0 ± 34.5	136.2 ± 33.3	123.8 ± 10.3
	SLC30A8 rs1326663 T/T (n=2)	SLC30A8 rs1326663 C/T (n=14)	SLC30A8 rs1326663 C/C (n=15)	SLC30A8 rs1326663 T/T (n=2)	SLC30A8 rs1326663 C/T (n=14)	SLC30A8 rs1326663 C/C (n=15)
0 min	82.0 ± 4.1	83.5 ± 7.3	81.7 ± 6.0	81.9 ± 2.4	81.2 ± 7.0	80.4 ± 6.8
30 min	131.8 ± 14.5	133.7 ± 23.1	138.3 ± 27.3	125.4 ± 12.2	131.0 ± 22.2	136.3 ± 15.1
60 min	130.0 ± 45.3	136.8 ± 27.8	148.9 ± 32.5	151.9 ± 2.6	139.6 ± 26.3	158.5 ± 25.7
90 min	129.4 ± 35.9	132.2 ± 30.6	129.2 ± 24.5	140.3 ± 1.8	142.5 ± 32.9	149.7 ± 26.0
120 min	112.3 ± 11.7	109.3 ± 29.7	112.9 ± 23.5	123.0 ± 2.8	132.4 ± 35.3	137.1 ± 32.2
	ZFAND6 rs11634397 A/A (n=6)	ZFAND6 rs11634397 A/G (n=19)	ZFAND6 rs11634397 G/G (n=6)	ZFAND6 rs11634397 A/A (n=6)	ZFAND6 rs11634397 A/G (n=19)	ZFAND6 rs11634397 G/G (n=6)
0 min	81.5 ± 3.6	82.9 ± 6.9	82.4 ± 7.7	78.1 ± 4.3	81.9 ± 6.4	80.4 ± 8.8
30 min	141.0 ± 34.3	138.3 ± 22.5	122.8 ± 17.5	126.2 ± 22.0	138.1 ± 17.3	124.8 ± 15.0
60 min	132.6 ± 54.0	142.3 ± 20.7	148.5 ± 31.9	147.1 ± 22.0	156.5 ± 25.2	129.8 ± 27.2
90 min	117.0 ± 35.3	130.2 ± 23.4	145.4 ± 26.3	146.2 ± 19.9	149.7 ± 30.6	133.3 ± 28.2
120 min	100.1 ± 31.7	111.7 ± 20.3	120.7 ± 33.7	121.1 ± 40.9	136.0 ± 28.5	141.0 ± 36.6

[1] OGTT Glucose: blood glucose at baseline and 30 minute intervals after 75 g oral glucose tolerance test at study baseline (PreBR) and after (PostBR) 7 days of bed rest

Supplementary Table S4. Insulin response to 75 g oral glucose tolerance test (OGTT) before and after 7 days of bed rest by genotype.

	OGTT Insulin PreBR[1] (μU/mL; mean ± SD)			OGTT Insulin PostBR (μU/mL; mean ± SD)		
	TCF7L2 rs7903146 C/C (n=14)	TCF7L2 rs7903146 C/T (n=13)	TCF7L2 rs7903146 T/T (n=4)	TCF7L2 rs7903146 C/C (n=14)	TCF7L2 rs7903146 C/T (n=13)	TCF7L2 rs7903146 T/T (n=4)
0 min	4.7 ± 2.9	4.5 ± 2.6	8.1 ± 6.0	7.0 ± 5.2	4.7 ± 2.5	6.0 ± 3.0
30 min	49.4± 39.4	33.9 ± 15.6	54.1 ± 16.8	71.5 ± 97.2	36.9 ± 29.0	63.9 ± 25.1
60 min	46.4 ± 41.0	38.8 ± 22.7	63.6 ± 21.2	67.9 ± 98.0	53.1 ± 49.8	73.0 ± 27.6
90 min	67.2 ± 76.9	39.9 ± 22.4	58.3 ± 31.8	71.9 ± 96.2	51.3 ± 19.9	70.4 ± 14.7
120 min	50.8 ± 47.4	28.4 ± 18.3	52.3 ± 35.4	75.4 ± 85.9	51.6 ± 20.8	63.0 ± 60.0
	MTNR1B rs10830963 C/C (n=15)	MTNR1B rs10830963 C/G (n=15)	MTNR1B rs10830963 G/G (n=1)	MTNR1B rs10830963 C/C (n=14)	MTN1RB rs10830963 C/G (n=13)	MTNR1B rs10830963 G/G (n=4)
0 min	4.6 ± 2.5	5.5 ± 4.2	4.4 ± 0	5.0 ± 2.7	6.8 ± 5.1	5.3 ± 0
30 min	42.1 ± 27.7	45.4 ± 32.9	36.4 ± 0	46.8 ± 24.7	66.0 ± 97.0	43.9 ± 0
60 min	40.2 ± 20.1	49.2 ± 41.9	66.6 ± 0	48.9 ± 20.6	76.6 ± 102.4	49.4 ± 0
90 min	45.0 ± 29.9	62.6 ± 73.5	78.2 ± 0	51.4 ± 20.5	74.7 ± 92.1	64.0 ± 0
120 min	42.0 ± 30.2	38.7 ± 43.6	78.2 ± 0	60.4 ± 26.8	66.9 ± 83.5	68.8 ± 0

	NOTCH2 rs1092393 G/G (n=26)	NOTCH2 rs1092393 G/T (n=4)	NOTCH2 rs1092393 T/T (n=1)	NOTCH2 rs1092393 G/G (n=26)	NOTCH2 rs1092393 G/T (n=4)	NOTCH2 rs1092393 T/T (n=1)
0 min	4.9 ± 3.4	4.5 ± 2.6	10.5 ± 0	5.5 ± 3.5	7.4 ± 6.9	11.3 ± 0
30 min	45.8 ± 31.6	30.3 ± 7.2	36.3 ± 0	58.8 ± 75.0	37.6 ± 18.3	57.1 ± 0
60 min	46.5 ± 35.0	36.3 ± 8.9	53.4 ± 0	65.3 ± 78.9	40.8 ± 16.4	72.8 ± 0
90 min	54.7 ± 59.6	51.3 ± 25.7	63.4 ± 0	64.0 ± 71.1	51.5 ± 19.3	86.8 ± 0
120 min	41.3 ± 39.2	38.9 ± 26.7	59.5 ± 0	64.5 ± 64.6	49.4 ± 19.7	104.6 ± 0
	RASGRP1 rs74035 C/C (n=21)	RASGRP1 rs74035 C/T (n=7)	RASGRP1 rs74035 T/T (n=3)	RASGRP1 rs74035 C/C (n=21)	RASGRP1 rs74035 C/T (n=7)	RASGRP1 rs74035 T/T (n=3)
0 min	5.3 ± 3.7	4.7 ± 2.9	3.9 ± 0.5	6.1 ± 4.0	6.1 ± 5.2	4.2 ± 0.6
30 min	46.6 ± 34.2	33.9 ± 13.8	43.9 ± 16.9	62.9 ± 81.9	31.2 ± 17.3	65.7 ± 29.7
60 min	51.3 ± 37.4	31.4 ± 11.1	37.0 ± 10.9	73.6 ± 85.9	33.7 ± 15.3	50.4 ± 22.3
90 min	61.3 ± 65.1	42.4 ± 22.7	35.6 ± 8.3	70.3 ± 78.5	47.1 ± 13.9	49.5 ± 21.4
120 min	45.1 ± 42.9	37.9 ± 20.4	25.5 ± 13.8	69.9 ± 71.2	53.5 ± 15.4	45.2 ± 37.4
	PROX1 rs2075423 T/T (n=6)	PROX1 rs2075423 G/T (n=11)	PROX1 rs2075423 G/G (n=14)	PROX1 rs2075423 T/T (n=6)	PROX1 rs2075423 G/T (n=11)	PROX1 rs2075423 G/G (n=14)
0 min	3.9 ± 1.0	6.4 ± 4.7	4.4 ± 2.5	5.7 ± 2.9	7.1 ± 5.4	5.1 ± 3.1
30 min	32.7 ± 8.9	58.2 ± 42.8	36.6 ± 16.8	39.1 ± 24.2	77.3 ± 109.7	46.5 ± 30.3
60 min	37.1 ± 19.1	53.3 ± 45.1	42.7 ± 24.7	48.7 ± 35.8	80.2 ± 110.1	54.2 ± 44.2
90 min	36.5 ± 22.2	74.1 ± 86.4	47.0 ± 23.1	50.6 ± 17.8	78.4 ± 108.5	56.4 ± 20.2

120 min	27.9 ± 26.8	58.6 ± 50.7	34.1 ± 22.2	54.6 ± 25.5	84.5 ± 93.7	51.6 ± 27.4
	HHEX rs111187 T/T (n=4)	HHEX rs111187 C/T (n=13)	HHEX rs111187 C/C (n=14)	HHEX rs111187 T/T (n=4)	HHEX rs111187 C/T (n=13)	HHEX rs111187 C/C (n=14)
0 min	4.0 ± 2.3	6.2 ± 4.3	4.2 ± 2.3	3.6 ± 2.1	7.1 ± 4.1	5.5 ± 4.2
30 min	47.9 ± 17.2	57.0 ± 38.8	29.7 ± 12.4	56.0 ± 44.4	80.9 ± 98.6	32.9 ± 19.7
60 min	64.1 ± 36.9	54.2 ± 40.2	31.9 ± 14.8	83.1 ± 78.8	84.8 ± 99.2	35.6 ± 16.9
90 min	69.2 ± 27.1	68.2 ± 79.2	37.8 ± 22.0	60.8 ± 27.7	79.5 ± 98.5	48.5 ± 16.4
120 min	40.2 ± 35.6	55.3 ± 46.6	29.3 ± 22.3	53.9 ± 23.6	82.3 ± 87.3	49.5 ± 23.7
	IGF2BP2 rs440296 G/G (n=11)	IGF2BP2 rs440296 G/T (n=17)	IGF2BP2 rs440296 T/T (n=3)	IGF2BP2 rs440296 G/G (n=11)	IGF2BP2 rs440296 G/T (n=17)	IGF2BP2 rs440296 T/T (n=3)
0 min	5.4 ± 4.3	4.4 ± 2.6	7.4 ± 3.1	5.3 ± 3.0	5.8 ± 4.1	8.8 ± 6.8
30 min	39.6 ± 18.0	40.3 ± 27.5	75.9 ± 60.3	47.4 ± 26.3	43.0 ± 29.4	161.5 ± 206.6
60 min	41.0 ± 13.8	37.1 ± 23.5	108.9 ± 59.5	50.7 ± 32.1	50.0 ± 43.2	175.0 ± 195.3
90 min	38.9 ± 13.2	45.6 ± 29.8	162.6 ± 134.3	52.4 ± 17.6	49.2 ± 21.4	180.8 ± 190.0
120 min	27.4 ± 12.9	37.9 ± 29.5	114.9 ± 58.0	57.5 ± 19.3	48.9 ± 27.9	171.8 ± 162.4
	CDKAL1 rs775484 G/G (n=10)	CDKAL1 rs775484 C/G (n=19)	CDKAL1 rs775484 C/C (n=2)	CDKAL1 rs775484 G/G (n=10)	CDKAL1 rs775484 C/G (n=19)	CDKAL1 rs775484 C/C (n=2)
0 min	3.6 ± 1.5	5.3 ± 3.7	9.9 ± 1.0	4.4± 3.0	6.2 ± 3.9	10.9 ± 8.0
30 min	27.5 ± 7.8	45.8 ± 26.1	102.1 ± 61.0	25.9 ± 11.3	54.4 ± 28.3	221.0 ± 253.2

60 min	30.4 ± 15.8	46.7 ± 22.8	107.8 ± 97.8	29.8 ± 12.8	63.7 ± 41.6	212.1 ± 265.7
90 min	36.1 ± 18.1	50.6 ± 29.1	184.4 ± 188.2	44.6 ± 16.6	56.0 ± 21.1	223.0 ± 250.3
120 min	23.0 ± 20.9	44.2 ± 27.6	109.9 ± 101.6	40.4 ± 21.0	60.6 ± 25.1	212.2 ± 207.6
	SLC30A8 rs1326663 T/T (n=2)	SLC30A8 rs1326663 C/T (n=14)	SLC30A8 rs1326663 C/C (n=15)	SLC30A8 rs1326663 T/T (n=2)	SLC30A8 rs1326663 C/T (n=14)	SLC30A8 rs1326663 C/C (n=15)
0 min	2.0 ± 0	5.4 ± 2.8	5.1 ± 3.9	3.3 ± 0.6	6.3 ± 4.4	5.9 ± 4.0
30 min	26.7 ± 5.0	45.0 ± 28.1	44.4 ± 32.9	36.9 ± 8.2	46.9 ± 24.6	67.0 ± 96.9
60 min	29.6 ± 13.4	42.5 ± 16.5	50.2 ± 43.6	45.5 ± 5.8	43.7 ± 21.6	82.0 ± 100.5
90 min	36.5 ± 26.4	51.0 ± 29.9	60.3 ± 74.4	54.7 ± 21.8	48.0 ± 19.9	78.3 ± 91.1
120 min	29.4 ± 6.0	42.2 ± 33.3	42.6 ± 43.1	57.8 ± 9.0	55.7 ± 28.1	72.2 ± 82.7
	ZFAND6 rs11634397 A/A (n=6)	ZFAND6 rs11634397 A/G (n=19)	ZFAND6 rs11634397 G/G (n=6)	ZFAND6 rs11634397 A/A (n=6)	ZFAND6 rs11634397 A/G (n=19)	ZFAND6 rs11634397 G/G (n=6)
0 min	2.6 ± 0.7	5.7 ± 3.7	5.4 ± 2.9	3.0 ± 0.9	7.1 ± 4.6	5.2 ± 1.6
30 min	32.0 ± 15.4	48.1 ± 35.2	40.3 ± 16.0	32.8 ± 27.1	68.4 ± 85.3	40.0 ± 14.0
60 min	26.0 ± 5.3	50.6 ± 37.7	48.6 ± 23.3	34.5 ± 13.7	78.1 ± 89.2	40.4 ± 20.6
90 min	29.5 ± 9.5	61.9 ± 68.1	56.5 ± 21.1	40.3 ± 10.3	72.8 ± 81.8	55.1 ± 21.9
120 min	19.5 ± 10.5	45.8 ± 43.1	50.5 ± 25.3	29.1 ± 15.7	75.4 ± 72.2	62.1 ± 25.3

[1] OGTT Insulin: Serum insulin at baseline and 30 minute intervals after 75 g oral glucose tolerance test at study baseline (PreBR) and after (PostBR) 7 days of bed rest

Supplementary Table S5: Description of intervention arms used in parent randomized controlled trial

Study Intervention	Description	PreBR 2hr OGTT[1] (mg/dL)	PostBR 2hr OGTT (mg/dL)
Skew	protein intake skewed toward dinner	92.6 ± 23.8	111.6 ± 26.4
Control	even protein distribution; received 0.06g/alanine/kg body weight at each meal	113.0 ± 31.6	149.3 ± 10.9
Leucine	even protein distribution; received 0.06g leucine/kg body weight at each meal	115.6 ± 31.6	136.1 ± 35.3
Whey	even protein distribution; whey protein isolate provided a majority of the protein at each meal	115.7 ± 22.3	131.9 ± 37.3
Step	even protein distribution; received 0.06 g alanine/kg body weight at each meal; walked 2000 steps during each day of bed rest	138 ± 0	147.5 ± 0

[1] 2hr OGTT: Blood glucose 2 hours after 75 g oral glucose tolerance test at study baseline (PreBR) and after (PostBR) 7 days of bed rest

Supplementary Table S6. Single Nulceotide Polymorphisms (SNP) included in the analysis.

Gene (or proximal gene)	SNP[1]	Position	Location	Minor, Major alleles	T2D[2] risk allele	MAF[3] (TOP MED)	Study MAF	HWE[4] p-value
MTNR1B	rs10830963	chr11:92975544	Intron	G, C	G	0.222	0.275	0.383
NOTCH2	rs10923931	chr1:119975336	Intron	T, G	T	0.165	0.097	0.233
RASGRP1	rs7403531	chr15:38530704	Intron	T, C	C	0.239	0.210	0.092
PROX1	rs2075423	chr1:213981376	Intergenic	T, G	G	0.343	0.371	0.245
HHEX	rs1111875	chr10:92703125	Intergenic	T, C	C	0.372	0.339	0.700
IGF2BP2	rs4402960	chr3:185793899	Intron	T, G	T	0.376	0.371	0.457
CDKAL1	rs7754840	chr6:20661019	Intron	C, G	C	0.401	0.371	0.132
SLC30A8	rs13266634	chr8:117172544	Missense	T, C	C	0.243	0.290	1.000
ZFAND6	rs11634397	chr15:80139880	Intergenic	G, A	G	0.457	0.500	0.295
TCF7L2	rs7903146	chr10:112998590	Intron	C, T	T	0.271	0.339	0.700

[1] SNP: single nucleotide polymorphism

[2] T2D: type 2 diabetes.

[3] MAF: minor allele frequency

[4] HWE: Hardy-Weinberg equilibrium

Supplementary Table S7. Single Nulceotide Polymorphisms (SNP) Allele Frequencies by Race

Gene (or proximal gene)	SNP[1]	Minor, Major alleles	T2D[2] risk allele	MAF[3] (TOP MED)	Study MAF	Asian MAF (n=2)	Black MAF (n=4)	Caucasian MAF (n=21)	Hispanic MAF (n=4)
MTNR1B	rs10830963	G, C	G	0.222	0.275	0.5	0.125	0.3095	0.125
NOTCH2	rs10923931	T, G	T	0.165	0.097	0	0.25	0.07	0.125
RASGRP1	rs7403531	T, C	C	0.239	0.210	0	0.375	0.165	0.375
PROX1	rs2075423	T, G	G	0.343	0.371	0	0.125	0.45	0.375
HHEX	rs1111875	T, C	C	0.372	0.339	1	0.375	0.26	0.375
IGF2BP2	rs4402960	T, G	T	0.376	0.371	0.5	0.625	0.335	0.25
CDKAL1	rs7754840	C, G	C	0.401	0.371	0.25	0.5	0.355	0.375
SLC30A8	rs13266634	T, C	C	0.243	0.290	0	0.25	0.355	0.125
ZFAND6	rs11634397	G, A	G	0.457	0.500	0.25	0.375	0.55	0.5
TCF7L2	rs7903146	C, T	T	0.271	0.339	0.25	0.5	0.3095	0.375

[1] SNP: single nucleotide polymorphism

[2] T2D: type 2 diabetes

[3] MAF: minor allele frequency

Supplementary Table S8. TCF7L2 Allele Frequencies.

P	N	rs7903146 Allele Freq	rs4506565 Allele Freq
Study[1]	31	C: 66.13%, T: 33.87%	A: 61.29%, T: 38.71%
AMR[2]	347	C: 76.51%, T: 23.49%	A: 74.06%, T: 25.94%
CEU[3]	99	C: 68.69%, T: 31.31%	A: 66.67%, T: 33.33%
MXL[4]	64	C: 78.12%, T: 21.88%	A: 75.0%, T: 25.0%
ASW[5]	61	C: 63.93%, T: 36.07%	A: 51.64%, T: 48.36%

[1] Current Study

[2] AMR: Ad Mixed American (National Cancer Institute (NCI) LDpop Tool, https://tinyurl.com/3p7tm6hk)

[3] CEU: Utah Residents (CEPH) with Northern and Western European Ancestry (NCI LDpop Tool, https://tinyurl.com/3p7tm6hk)

[4] MXL: Mexican Ancestry from Los Angeles USA (NCI LDpop Tool, https://tinyurl.com/3p7tm6hk)

[5] ASW: Americans of African Ancestry in SW USA (NCI LDpop Tool, https://tinyurl.com/3p7tm6hk)
